# Weak interactions in higher-order chromatin organization

**DOI:** 10.1093/nar/gkaa261

**Published:** 2020-04-20

**Authors:** Omar L Kantidze, Sergey V Razin

**Affiliations:** Institute of Gene Biology Russian Academy of Sciences, 119334 Moscow, Russia

## Abstract

The detailed principles of the hierarchical folding of eukaryotic chromosomes have been revealed during the last two decades. Along with structures composing three-dimensional (3D) genome organization (chromatin compartments, topologically associating domains, chromatin loops, etc.), the molecular mechanisms that are involved in their establishment and maintenance have been characterized. Generally, protein–protein and protein–DNA interactions underlie the spatial genome organization in eukaryotes. However, it is becoming increasingly evident that weak interactions, which exist in biological systems, also contribute to the 3D genome. Here, we provide a snapshot of our current understanding of the role of the weak interactions in the establishment and maintenance of the 3D genome organization. We discuss how weak biological forces, such as entropic forces operating in crowded solutions, electrostatic interactions of the biomolecules, liquid-liquid phase separation, DNA supercoiling, and RNA environment participate in chromosome segregation into structural and functional units and drive intranuclear functional compartmentalization.

## INTRODUCTION

Recent data suggest that the 3D genome organization plays an important role in the regulation of gene expression. The regulatory events occur at different levels of genome folding, starting from nucleosome positioning on DNA that interferes with transcription factors binding to their recognition sites on DNA. Still, when discussing 3D genome organization, most scientists mean the specific configuration of nucleosomal fiber (10 nm chromatin fiber) within the nuclear space, or, more specifically, within a chromosomal territory. Here, there are at least two levels of chromatin folding: chromatin loops, some of which bring promoters into the vicinity of enhancers (1), and partitioning of a chromosome into topologically associating domains (TADs) (2–4) that restrict the areas of enhancers’ action (5). Although many authors emphasize the importance of establishing a specific configuration of extended genomic segments for the transcription control, the relationship between the 3D organization of the genome and the implementation of functional processes is not that clear. A relationship between the 3D genome organization and genome functional activity has been addressed in several recently published reviews (6–10).

**Figure 1.**
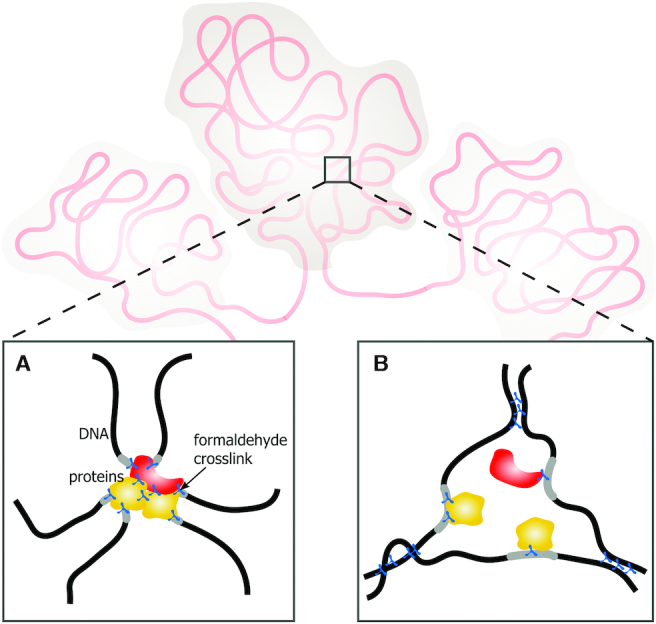
Active chromatin hub (ACH) model. (**A**) Chromatin hub as a rigid complex of regulatory elements stabilized by protein–DNA and protein–protein interactions. (**B**) Chromatin hub as a nuclear compartment. Black lines represent segments of chromatin fibers with regulatory elements shown in gray. Yellow and red figures represent transcription factors and transcription machinery proteins, respectively; blue figures indicate formaldehyde cross-links.

Systematic studies of the 3D genome began with the development of a 3C procedure based on a proximity ligation principle (11) and particularly with the development of Hi-C, a genome-wide derivative of the 3C procedure (12). The idea behind the original 3C protocol was quite simple (Figure 1A). If two distant DNA fragments situated at the base of chromatin loop interact with each other via proteins, the putative protein bridge can be fixed by formaldehyde. After lysis of the nucleus and fragmentation of a DNA fiber, the DNA fragments linked by a protein bridge will be preferentially ligated to each other if ligation is carried out at a low DNA concentration. Chimeric DNA fragments generated by this procedure will bear information about the spatial proximity of DNA fragments that were cross-linked via a proteinaceous bridge. Subsequent studies demonstrated that in the course of a 3C procedure, fixed nuclei survive SDS treatment, and hence proximity ligation proceeds within nuclei rather than in a diluted solution (13). Furthermore, the attempts to solubilize chromatin by mild sonication before performing a proximity ligation procedure resulted in a loss of information about the spatial proximity of distant DNA fragments (13). It was concluded that the maintenance of specific folding of large chromosomal segments within a cell nucleus in addition to the direct linkage of remote DNA regions by protein bridges is important for mediation of the proximity ligation (14) (Figure 1B). Consequently, in most of the recent studies, the so-called *in situ* (in-nucleus) Hi-C protocol is used (15–20).

Initial application of the 3C procedure for the analysis of the spatial proximity of various segments of the mouse β-globin gene domain demonstrated that distant regulatory elements and promoters of transcribed genes are located close to each other, possibly within the same DNA-protein complex termed the active chromatin hub (ACH) (21–23). Later similar observations were made in a number of other genomic loci (24–26). However, the nature of ACHs remained obscure. Recent evidence suggests that transcription factors and components of transcription machinery bound to an enhancer form an activating compartment via liquid-liquid phase separation (LLPS) (27–29). Promoters and enhancers share a number of interacting proteins including RNA polymerase II (Pol II) and Mediator complex subunits; these proteins possess intrinsically disordered domains and are capable of forming liquid phase condensates (30–32). Being juxtaposed in a nuclear space, the liquid compartments bound to enhancers and promoters may fuse to form a common compartment. Within this compartment, activation of transcription is likely achieved due to the high concentration of transcription factors and components of the transcription machinery (33). For our discussion, it is important that, in this scenario, the juxtaposition of enhancer and promoter is ensured by the specific configuration of chromatin fiber rather than by capturing an occasional colocalization. Indeed, when erythroid cells are placed into hypoosmotic stress conditions, the nuclei become expanded, and ACHs are disassembled as evidenced by a loss of juxtaposition of the components of the β-globin domain ACH (34). However, after returning to normal conditions, ACHs are rapidly reassembled. This process occurs even at low temperatures that disfavor biological processes and a search of partners by a random walk. It is thus likely that chromatin fiber possesses ‘a memory’ of initial configuration that is reestablished without the contribution of any biological processes (34).

The mode of chromatin folding at the levels above the nucleosomal fiber is still poorly understood. The modern idea is that in mammals, the final configuration of chromatin chain within a chromosomal territory is established by an interplay of two processes: active DNA loop extrusion by cohesion motors and passive segregation of the so-called compartmental domains bearing distinct chromatin marks (35–37). At a megabase resolution, the most evident feature of spatial chromatin organization is the segregation of active (‘A’) and repressed (‘B’) chromatin compartments (12). At 100 kb resolution in mammals and 10–20 kb resolution in Drosophila, one can observe partitioning of chromosomes into self-interacting domains termed topologically associating domains or TADs (2–4). It should be noted that the level of TADs insulation is rather moderate (38,39), and positions of TADs established by analysis of population data may vary in individual cells (39,40). Recent data suggest that, in mammals, TADs are generated by active DNA loop extrusion (41,42). This process coexists with segregation of relatively small compartmental domains and partially overrides the profile of these domains, which becomes more evident after blocking loop extrusion (43). There is no data demonstrating that DNA loop extrusion contributes to the spatial genome organization in Drosophila (35,36). In the Drosophila genome, TADs are likely generated purely by segregation of active and repressed chromatin domains (44). Segregation of compartmental domains, as well as compaction and shaping of chromatin loops, are directed by various physical forces, interactions, and processes such as electrostatic interactions, depletion attraction, or entropic forces operating under conditions of macromolecular crowding and LLPS. The final organization of a chromatin fiber in the nuclear space can be influenced by transcription-generated superhelical tension in DNA, the interaction of DNA with architectural proteins and RNAs, and the recruitment of certain genes to functional nuclear compartments. Each of the above interactions that we commonly refer to as ‘weak interactions’ may be insufficient to impact the chromatin folding, but together, they can contribute significantly to the shaping of chromatin fiber. Below, we discuss the contribution of different weak interactions into establishing and maintaining the 3D genome architecture.

## ELECTROSTATIC INTERACTIONS

**Figure 2.**
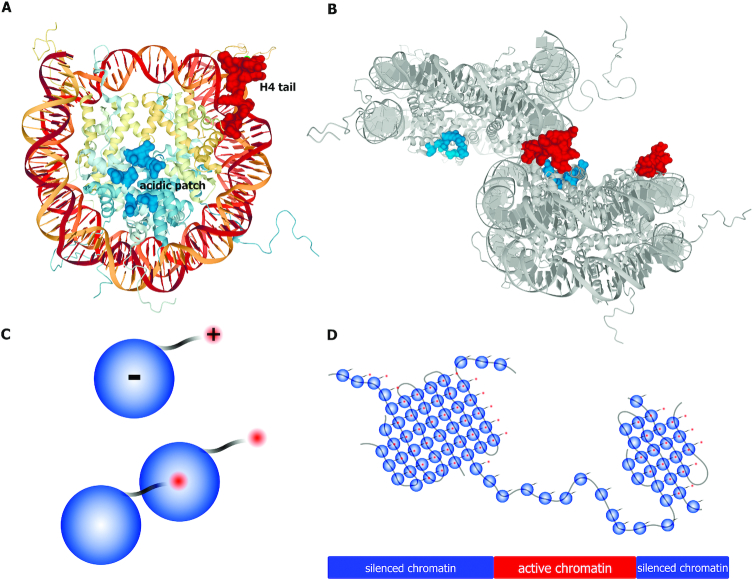
Electrostatic interactions can mediate nucleosome self-association. (**A**) Structure of the nucleosome (Protein Data Bank code: 1KX5) is viewed down the superhelical axis of the DNA. Histones H3, H4, H2A and H2B are shown in light blue and yellow colours. Acidic residues on H2A and H2B (the ‘acidic patch’) that are involved in interaction with the H4 tail and with nucleosome-interacting proteins (LANA peptide, interleukin-33, regulator of chromosome condensation 1, silent information regulator 3, and high mobility group nucleosome-binding domain-containing protein 2) are indicated as molecular surfaces in bright blue. Residues of the histone H4 tail are indicated as molecular surfaces in bright red. (**B**) Potential nucleosome–nucleosome interactions in the crystal structures of unconnected 147 bp nucleosomes (Protein Data Bank code 1KX5) mediated by histone H4 tail and acidic patch. (**C**) A scheme illustrating the ability of non-acetylated nucleosomes to establish spatial contacts via an interaction between a positively charged histone tail of one nucleosome and the negatively charged acidic patch of another nucleosome. Nucleosomes with acetylated histone tails lack such ability. (**D**) Model of chromatin partitioning into TADs/inter-TADs based on self-association of non-acetylated nucleosomes present within silenced chromatin regions.

The nucleosome core particle consists of histone octamer and wrapped DNA. Whereas the histone octamer as a whole has a positive charge, there is a local negatively charged area on the surface of the octamer, termed the acidic patch (Figure 2A) (45). Interaction of the positively charged histone tails, first of all the H4 histone tail, of one particle with the acidic patch on the other particle can keep particles together (Figure 2B-C) (46–48). These interactions have long been recognized as a driving force for the formation of the so-called 30 nm nucleosomal fibers or less regular nucleosomal aggregates depending on some additional conditions (49–52). Whatever the exact mode of chromatin folding is, it is certain that the electrostatic interactions of nucleosomal particles can play an essential role in this process (Figure 2D). Acetylation of histone tails reduces their positive charge and consequently interferes with electrostatic interactions of nucleosomal particles (53). Highly acetylated active chromatin is packed in a less compact manner compared to repressed chromatin (54,55). Recent studies do not produce evidence for the existence of extended stretches of nucleosomal chain organized into 30 nm fiber in living cells (56–59). However, electrostatic interaction between nucleosomes is likely to underlie the assembly of irregular supramolecular complexes of various sizes (60–62). It has been proposed that the interaction of non-acetylated nucleosomes of inactive chromatin underlies the assembly of TADs in Drosophila (44).

## LIQUID–LIQUID PHASE SEPARATION (LLPS)

**Figure 3.**
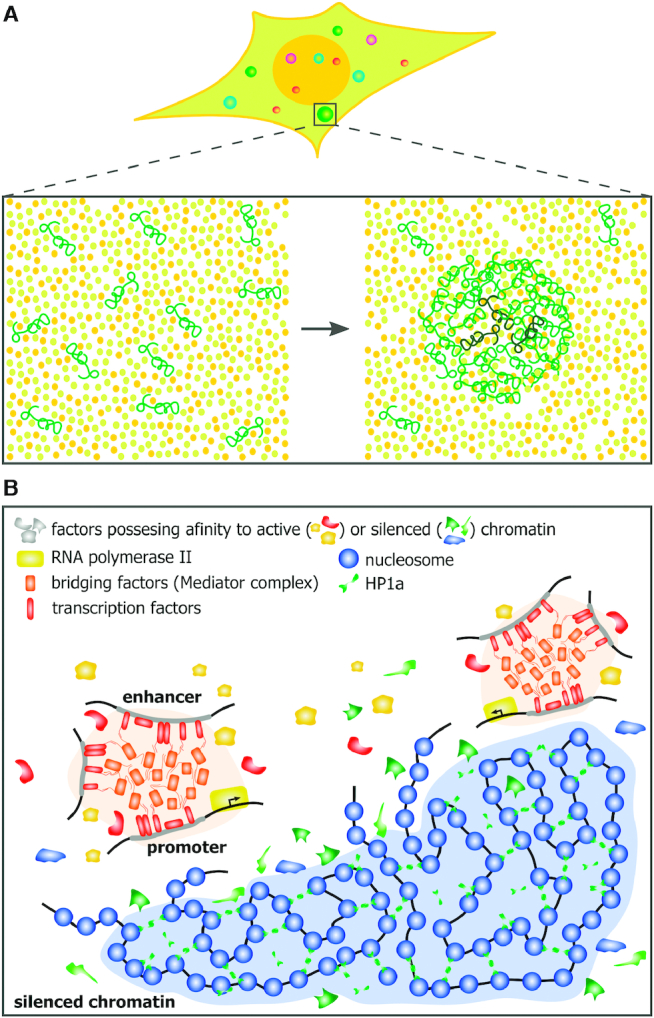
Liquid–liquid phase separation contributes to higher-order chromatin organization. (**A**) Schematic demonstrating how liquid-liquid phase separation underlies the formation of biomolecular condensates (i.e. cellular compartments that concentrate macromolecules without surrounding membranes). (**B**) LLPS-mediated transcription activating condensates (light orange) and constitutive heterochromatin domain (light blue).

Liquid–liquid phase separation is a process exemplified by the formation of oil droplets in an aqueous medium. In the cell nucleus, the main driving force of LLPS is interaction of intrinsically disordered domains (IDRs) present in many proteins involved in nuclear compartmentalization, chromatin compaction and nucleic acids metabolism (Figure 3A) (30,31,63–68). These IDRs may mediate weak-affinity and non-specific interactions with multiple target sites that trigger LLPS (69). Of note, histones H1 and H2A can form phase-separated liquid condensates *in vitro* in the presence of either DNA or nucleosomes (70,71). Furthermore, being microinjected into nuclei, reconstituted chromatin undergoes phase separation producing dense and dynamic droplets; this process is antagonized by acetylation of histone tails (71).

LLPS can contribute to the 3D genome organization in different ways. First, it may be involved in the generation of distinct chromatin compartments, and second, it underlies the assembly of functional nuclear compartments to which remote genomic elements are recruited. For several decades, it was generally accepted that heterochromatin is packed in such a dense manner that transcription factors and components of transcription machinery simply cannot reach DNA in heterochromatin (72). Recent data suggest that this is not the case because relatively large molecules can easily permeate through both euchromatic and heterochromatic regions (73). Furthermore, some essential genes are located in heterochromatin and are transcribed (74). Surprisingly, in Drosophila, the expression of heterochromatic genes is compromised in the euchromatic environment (75). Recent data provide an explanation for this enigma. Several lines of indirect evidence suggest that heterochromatin represents only a distinct chromatin compartment generated via LLPS (66–68,76). The essential feature of this compartment is that although many proteins can enter it only some of them are retained within this compartment (Figure 3B).

An assumption that heterochromatin domains are formed via LLPS explains many features of heterochromatin. Yet it still lacks direct experimental proves. Most of the arguments in favor of LLPS-mediated heterochromatin assembly are obtained in experiments *in vitro*. Meanwhile, heterochromatin clusters present within the cell nuclei do not possess all expected features of liquid condensates (77). For example, these domains do not necessarily have round shape, are not easily disrupted by 1,6-hexandiol treatment, and their crucial components, such as HP1, do not rapidly exchange with the nucleoplasmic pull. It is thus possible that being initially assembled via LLPS heterochromatic domains eventually undergo gelation (76,78). In some cases, correct interpretation of the experimental observations pointing to the involvement of LLPS in the assembly of various biological structures may only be done if quantitative characteristics are taken into consideration (79,80).

It has been argued that polymer-polymer phase separation explains the properties of heterochromatin much better than LLPS (81), and results of a recent study of mouse heterochromatin demonstrate that chromocenters represents collapsed chromatin globules formed via polymer-polymer phase separation rather than LLPS-derived liquid droplets (77).

Another way the LLPS can shape the 3D genome is an assembly of functional nuclear compartments and retention of distal genomic elements within these compartments. Thus, Pol II, Mediator, and many transcription factors possess IDRs (30,31,82,83) that are capable of interacting with each other, triggering LLPS. Multi-bromodomain proteins attracted to acetylated H3 tails may also contribute to LLPS in active genomic regions (71). Recent data demonstrate that the formation of phase-separated liquid activating domains at enhancers is a pre-requisite for transcription activation by enhancers (Figure 3B) (27,84,85). To be activated by remote enhancers, promoters should be placed within such domains, a positioning that is possible only at the level of the 3D genome via looping of an intervening segment of DNA fiber (86,87). Phase-separated liquid compartments are assembled both on enhancers (27,84,85) and promoters (30). The fusion of these compartments would keep an enhancer and target close to each other and thus stabilize chromatin loops, whatever is the mechanism that initially brings an enhancer and a target promoter into spatial proximity. It should be noted that, within the activating compartment, enhancers and promoters may be transiently bridged via proteins, such as Mediator (88) or some transcription factors (89,90).

It has long been reported that transcribing RNA polymerases are assembled into clusters termed transcription factories (reviewed in (91–93). Recent data suggest that these factories are dynamic and are assembled via LLPS (30,94,95). The principles of genes’ assembly into transcription factories are poorly understood (93). Some studies demonstrate that closely located genes are assembled in transcription factories independently of their tissue specificity (96,97); another provides evidence for the existence of tissue-specific transcription factories (98). In any case, the recruitment of remote genes to the same transcription factory should drastically affect the 3D genome and hence should be considered to be an important factor of spatial genome organization. Besides transcription factories, remote genes can be attracted to Cajal bodies (99) and nuclear speckles (100–103) that both represent functional nuclear compartments formed by LLPS (104,105). The association of active genes with nuclear speckles was reported to have a significant impact on spatial segregation of active and repressed chromatin compartments (106,107).

## MACROMOLECULAR CROWDING

Macromolecular crowding is a physicochemical phenomenon that occurs in concentrated solutions of macromolecules when macromolecules occupy 20–30% of the total volume. Under these conditions, entropic or ‘depletion attraction’ forces promote the aggregation of macromolecules (108–112). This phenomenon can be explained based on the way in which macromolecules interact with each other and molecules of the solvent. In solution, macromolecules are constantly bombarded by smaller molecules of solvent undergoing Brownian movement. Macromolecules also move, and once they happen co-contact each other, the pressure of smaller molecules will keep them in proximity to each other; the molecules of solvent will impact them from the outside, but there is no force that would push them from the inside or pull them in the outward direction. The aggregation of macromolecules in concentrated solutions is thermodynamically favorable because it increases the volume accessible to small molecules and thus causes a gain in entropy. Within a cell, macromolecular crowding is generated by large biomolecules, such as proteins, nucleic acids, and polymeric carbohydrates (113). In model experiments, an increase in the level of macromolecular crowding caused by the addition of crowding agents promotes the aggregation of chromatin fibers and chromatin compaction *in vitr*o (114,115) and *in vivo* (112,116). Conditions of macromolecular crowding are typical for the cytoplasm and nucleoplasm. In the cell nucleus, entropic forces stabilize various nuclear compartments such as nucleoli, Cajal bodies and ND10 bodies (110,117,118). It should be mentioned that entropic forces would stabilize macromolecular aggregates of any kind. The nature of any particular compartment, including chromatin compartments, would be determined by additional conditions including mutual affinity of components constituting this compartment. Nuclear compartments, including active chromatin hubs (34) and various nuclear bodies (110,117) are easily disassembled under hypoosmotic shock and reassembled upon the addition of a crowding agent in a hypoosmotic medium (34,110,117). Importantly, reassembled compartments retain their identity (do not mix). Hence, the nature of compartments is determined by specific interactions, rather than entropic forces.

## DNA SUPERCOILING

In eukaryotic chromatin, DNA wrapped around nucleosomal core particles is negatively supercoiled (45). The DNA supercoils on nucleosomes are referred to as being constrained because they cannot be relaxed by topoisomerases. Besides this, synthesis processes such as replication and transcription generate the supercoiling in unconstrained internucleosomal linkers. Although both replication and transcription machinery can generate supercoiling, the most physiologically relevant DNA supercoiling in eukaryotes is produced by transcription, which generates positive supercoiling ahead and negative supercoiling behind an RNA polymerase (119,120). The DNA torsion that develops is relieved by the action of type I and II topoisomerases (121). Because an overwound state can hinder DNA replication and transcription, positive supercoiling is eliminated much more rapidly than negative supercoiling (122). By facilitating DNA melting and stabilization of alternative DNA structures such as R-loops (123), Z-DNA (124), cruciform (125), and G quadruplexes, negative supercoiling clearly contributes to the regulation of gene expression (126). Positive supercoiling also induces nucleosome eviction due to the unwinding of DNA (119,120). The resultant enhanced flexibility of the chromatin fiber can promote its spatial exploration and, thus, the establishment of remote *in trans* interactions. Supercoiling has long been acknowledged as a molecular force associated with the higher-order chromatin organization based on the early observations that type II DNA topoisomerases are often bound to chromatin at DNA loop basements (127–129). However, the causative role of DNA supercoiling in higher-order chromatin organization remains elusive.

**Figure 4.**
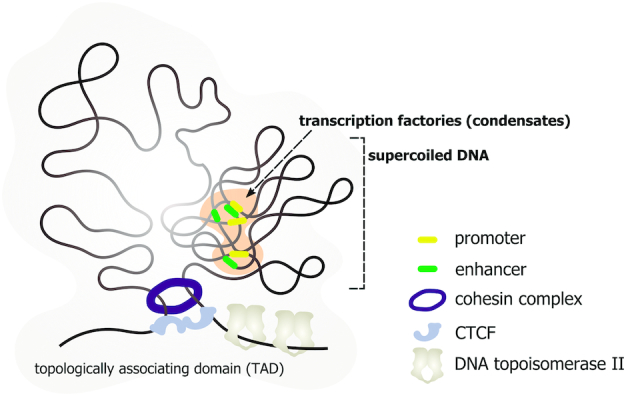
DNA supercoiling is one of the forces that increase the efficiency of intra-TAD promoter–enhancer communications. Low-level transcription of enhancers can lead to accumulation of negative supercoiling in particular intra-TAD regions that increases the frequency of the contact events between distant regulatory elements, promoters, and enhancers.

The presence of unconstrained DNA supercoils in chromatin has been discussed for years (119,130–135). However, the research tools that allow genome-wide analyses of unconstrained DNA supercoiling in chromatin have only recently been developed (136,137). Using psoralen intercalation-based approaches, it was shown that transcription-dependent negative supercoiling was enhanced near transcription start sites (TSSs) (138,139) and that human interphase chromosomes are partitioned into domains with different levels of supercoiling (140). By number, more than half of such domains were underwound with approximately 15% and 35% of overwound and stable ones, correspondingly (140). The negative supercoiling domains quite accurately coincide with actively transcribed regions and with the sites of chromatin-bound DNA topoisomerase II (140,141). However, the supercoiling domains do not correspond directly to the chromatin compartments and TADs (140–142). The supercoiling domains have a median size of 100 kb (140), which likely relates them to the chromatin loops. Based on molecular dynamics simulations, it was proposed that transcription-generated supercoiling could contribute to the loop extrusion process—in this case, supercoiling had been considered to be a molecular force that moves a cohesin ring along the DNA (143,144). This mechanism might, particularly, explain the colocalization of CTCF sites at the boundaries of loop-forming TADs with the chromatin-bound DNA topoisomerase II, which is presumably required to relieve DNA tension accumulated inside the specific TAD (141,145). However, a particularly recent discovery of cohesin's DNA translocase activity (146,147), and the fact that the establishment of loop domains does not require transcription and replication (148), compromise this hypothesis. Furthermore, due to the substitution of histones by protamines, transient DNA nicks, and the lack of active transcription, sperm DNA is characterized by a decreased level of the supercoiling (149–151). At the same time, the spatial genome organization of sperm DNA is virtually the same as the ones of somatic or stem cells (152,153). These observations unequivocally question the importance of DNA supercoiling for the establishment and maintenance of basic higher-order chromatin organization in mammals. However, this does not exclude a possibility that supercoiling contributes to the shaping of the internal structure of TADs. The most accurate molecular dynamics simulations showed that the supercoiling of the TADs could significantly increase the efficiency of intra- versus inter-TAD enhancer-promoter communications (154,155). Being in good agreement with the experimental data, this observation supports the essential role of supercoiling in genome organization and gene expression (Figure 4). From this point of view, transcription of enhancers can be considered to be an initial producer of supercoiling, which drives enhancer-promoter communications (144).

Although there is clear evidence for the role of active DNA loop extrusion in mammalian TAD formation (41,42,148,156), the mechanisms underlying the formation of contact chromatin domains in other taxa remain less clear. Molecular dynamics simulations show that, in fission yeast, a superhelical tension introduced into DNA by transcribing RNA polymerases moving in a convergent direction may be sufficient to explain the formation of contact chromatin domains (157).

## RNA ENVIRONMENT

The vast majority of the genome is transcribed to generate heterogeneous nuclear RNAs (hnRNAs) that include Pol II-dependent coding, noncoding and regulatory RNAs (158,159). Only 5% of hnRNAs reach the cytoplasm (160). Taken together with the RNA polymerase I-mediated transcripts, hnRNAs comprise an essential part of the nucleoplasm. Thus, it is tempting to suggest that RNAs retained in the nucleus somehow contribute to the nuclear structure and dynamics as well as higher-order chromatin organization (161). Indeed, there are several examples of how the specific RNAs participate in the establishment and maintenance of chromatin states and genome organization (reviewed in (162)). It is well defined how the XIST RNA mediates topological reconfiguration of inactivated X (Xi) chromosome in female cells, and how the FIRRE RNA promotes trans-chromosomal interactions of the Xi (163–166). Asynchronous replication and autosomal RNAs (ASARs) involved in the establishment of monoallelic expression represent another example of architectural RNAs that coat extended chromosomal regions (167,168). Specific noncoding RNA species are critical for the assembly of various subnuclear compartments (nuclear bodies) providing a platform for their assembly (169). However, these observations do not provide a clear explanation of how bulk RNAs contribute to the 3D genome organization. Recent genome-wide studies of RNA–DNA interactions have demonstrated that much of the genome is covered with coding and noncoding RNAs (170–173). While most of such chromatin-associated RNAs (caRNAs) bind DNA in *cis* (i.e. at sites of their synthesis), some are known to bind DNA in *trans* (172–173). The coding sequences for some of the long noncoding RNAs often coincide with the CTCF binding sites and TAD boundaries that implicitly indicates their role in spatial genome organization (174,175). The possible mechanistic ways in which this chromatin-associated RNA cloud participate in higher-order chromatin organization have been revealed only recently. First, a known RNA-binding activity of CTCF is essential for chromatin loop formation (176,177). It was shown that approximately half of all CTCF-dependent loops was disrupted in cells expressing CTCF lacking its RNA-binding region (176). These findings highlight the functional role of the caRNAs, particularly noncoding RNAs that are transcribed near the TAD boundaries. Second, a scaffold attachment factor A (SAF-A), also referred to as heterogeneous ribonucleoprotein U (HNRNP-U), which can bind to most RNA species, was shown to participate in the maintenance of the 3D genome (178,179). Specifically, it was demonstrated that depletion of the SAF-A results in compartment switching on 7.5% of the genome, in decreased TAD boundary strengths, and in reduced chromatin loop intensities (178). Mechanistically, this is achieved through an ATP-dependent oligomerization of SAF-A with caRNAs that results in chromatin mesh modulating large-scale chromosome structures (179). Another nuclear matrix protein, the scaffold attachment factor B (SAFB), also contributes to spatial genome organization; in particular, it maintains the higher-order organization of pericentromeric heterochromatin (180). SAFB interacts with heterochromatin-associated repeat transcripts (major satellite RNAs) that promote SAFB-driven phase separation of the heterochromatin compartment (180). One of the intrinsic properties of the IDR-containing proteins, which often drive phase separation, is their RNA-binding activity (181). Chromatin-associated RNAs might thus serve as nucleation centers for an LLPS-mediated 3D genome reconfiguration. In this scenario, transcription would act as a tunable switch for this process. In the same manner, a functional role of the RNAs transcribed from enhancer sequences (enhancer RNAs, eRNAs) in facilitating enhancer-promoter interactions can be interpreted (182–184). Transcription of the eRNAs can initiate the Mediator-driven LLPS to form an enhancer-promoter loop (32,185,186).

It was shown recently that caRNAs could influence chromatin organization by counteracting histone electrostatic interactions (187). Negatively charged caRNAs neutralize the charge of histone tails upon binding to chromatin and, thus, reduce electrostatic compaction of DNA (187). This effect depends on single-stranded nature of RNA, its length, concentration, and negative charge, but not on its specific sequence. Particularly, LINE1 RNA binds histones and potentially utilizes the described mechanism for maintaining an open chromatin state (187). Nevertheless, the full spectrum of RNA types involved in this mechanism of chromatin state regulation is not identified.

## CONCLUDING REMARKS

The recent advances in verifying the DNA loop extrusion model of mammalian TAD formation have drawn attention away from other possible mechanisms of TAD assembly. Having no intention to question the value of the DNA loop extrusion model, we still wish to outline the facts that are difficult to explain in frames of this model. First, TAD-like contact chromatin domains have been observed in various taxa (4,188–191), whereas all arguments for active DNA loop extrusion have so far been obtained in mammals only. Second, the DNA loop extrusion model considers TADs to be a population phenomenon originating due to a superimposition of various looped domains occurring at a specific timepoint in individual cells (41,42). Meanwhile, distinct chromatin globules colocalizing with TADs annotated on Hi-C maps have been observed in individual cells using various FISH-based protocols (192–195). The recently developed multiplex FISH-based approaches allow tracing chromatin conformation at kilobase-scale resolution (193–195). The single-cell chromatin interaction maps obtained using these approaches demonstrate presence of self-interacting globular domains. Although positions of domains boundaries varied between individual cells, they were preferentially located in regions bound by CTCF and cohesin (193). Third, TAD-sized self-interacting chromatin domains were observed in individual human cells lacking a functional cohesin complex (193). However, the specific positioning of these domains was lost (193). The authors of the above-cited study concluded that even in mammals, the DNA loop extrusion imposes some constraints on the positioning of TAD-like domains rather than contributing to their assembly (193).

**Figure 5.**
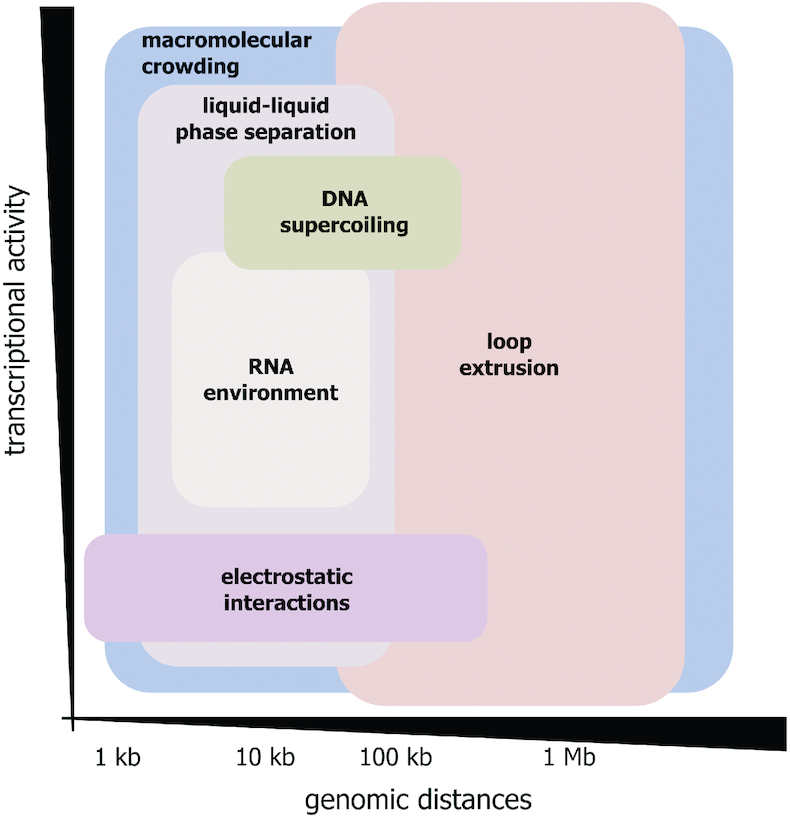
Relative contribution of weak interactions discussed and loop extrusion to spatial genome organization in higher eukaryotes depending on genomic distances and transcriptional activity of genomic regions. Macromolecular crowding being a general physico-chemical phenomenon seems to contribute to the folding of chromatin at all structural levels (genomic distances) irrespective of transcriptional activity of genomic regions. The existing evidence suggests that all other weak biological forces discussed here (LLPS, DNA supercoiling, electrostatic interactions, and RNA environment) mostly influence genome organization at a kilobase-scale. While LLPS and RNA environment can contribute to the folding of both transcriptionally active and inactive genomic regions, histone electrostatic interactions are involved in the spatial organization of transcriptionally silenced chromatin, and the influence of DNA supercoiling is generally restricted to the transcriptionally active sites.

We suggest that the weak forces discussed in this review contribute altogether, although to various extents, into an assembly of contact chromatin domains (Figure 5). The basic mechanism of the assembly of chromatin globules is likely to involve condensation of nucleosomes directed by electrostatic interactions (46,51,52). This process should be particularly relevant in the case of inactive chromatin domains (44) because a high level of histone acetylation, which is typical for active chromatin (196), suppresses the electrostatic interactions of nucleosomes (53,197,198). Condensation of nucleosomes may represent a main driving force of TAD assembly, as occurs in Drosophila (44) or may complement the work of other mechanisms. For example, it may compact extruded DNA loops. Condensation of nucleosomes is likely to be shaped and stabilized via LLPS. The latter, in turn, would depend on the interaction of specific sets of proteins and possibly also regulatory RNAs with certain genomic regions. In the simplest case, recruitment of HP1 to domains of H3K9me3 may cause LLPS (67) or even gelation (199). LLPS is likely to contribute to establishing long-range inter- and intrachromosomal contacts via an assembly of transcriptional factories, active chromatin hubs, and various nuclear bodies to which remote genomic elements become recruited in connection with a realization of various functional processes.

Recent evidence suggests that both active and repressed genomic regions are organized into contact chromatin domains, although of different sizes (15,19,35). In active chromatin, electrostatic interaction of nucleosomes can hardly play an important role due to the high level of histone acetylation. In contrast, superhelical tension introduced by transcription may contribute to the compaction of active genome regions (143).

Although all of the above-discussed interactions are rather weak, their cumulative effect is likely to be substantial. Furthermore, any kind of molecular condensates, including globular chromatin domains, should be additionally stabilized by entropic (depletion-attraction) forces operating in the crowded nuclear milieu.

What is the role of DNA loop extrusion in the above-described scenario? It has been suggested that this is a relatively new evolutionary acquisition that appeared only in vertebrates (35,36). DNA loop extrusion operates simultaneously with more basic mechanisms. It does not interfere with the partitioning of the chromosomes into segregated epigenetic domains but partially overwrites them (43) to divide chromosomes into regulatory domains that restrict the areas of enhancer action (5,200). As stated above, the extruded chromatin loops may be further compacted and stabilized by mechanisms discussed in this review.
